# S100A9 Interaction with TLR4 Promotes Tumor Growth

**DOI:** 10.1371/journal.pone.0034207

**Published:** 2012-03-28

**Authors:** Eva Källberg, Thomas Vogl, David Liberg, Anders Olsson, Per Björk, Pernilla Wikström, Anders Bergh, Johannes Roth, Fredrik Ivars, Tomas Leanderson

**Affiliations:** 1 Immunology Group, Lund University, Lund, Sweden; 2 Institute of Immunology, University of Münster, Münster, Germany; 3 Active Biotech AB, Lund, Sweden; 4 Department of Medical Bioscience, Umeå University, Umeå, Sweden; Enzo Life Sciences, Inc., United States of America

## Abstract

By breeding TRAMP mice with S100A9 knock-out (S100A9^−/−^) animals and scoring the appearance of palpable tumors we observed a delayed tumor growth in animals devoid of S100A9 expression. CD11b^+^ S100A9 expressing cells were not observed in normal prostate tissue from control C57BL/6 mice but were readily detected in TRAMP prostate tumors. Also, S100A9 expression was observed in association with CD68^+^ macrophages in biopsies from human prostate tumors. Delayed growth of TRAMP tumors was also observed in mice lacking the S100A9 ligand TLR4. In the EL-4 lymphoma model tumor growth inhibition was observed in S100A9^−/−^ and TLR4^−/−^, but not in RAGE^−/−^ animals lacking an alternative S100A9 receptor. When expression of immune-regulating genes was analyzed using RT-PCR the only common change observed in mice lacking S100A9 and TLR4 was a down-regulation of TGFβ expression in splenic CD11b^+^ cells. Lastly, treatment of mice with a small molecule (ABR-215050) that inhibits S100A9 binding to TLR4 inhibited EL4 tumor growth. Thus, S100A9 and TLR4 appear to be involved in promoting tumor growth in two different tumor models and pharmacological inhibition of S100A9-TLR4 interactions is a novel and promising target for anti-tumor therapies.

## Introduction

The TRAMP prostate cancer model is an established spontaneous model of prostate cancer in immune competent mice. The mechanistic basis behind the model is a transgenic construct where the SV40 large antigen is expressed under control of the probasin promoter [1]. Some variations in the time of tumor incidence have been reported and appear to vary with the genetic background of the animals [2], but most animals develop a tumor before week 30. In addition, the TRAMP model has been used to study metastasis and changes in the immune function of animals at different stages of tumor development [3], [4].

MDSCs are a heterogeneous population of cells (reviewed in [5], [6],[7]) characterized by the expression of CD11b and Gr-1 in the mouse [8] [9]. These cells are strongly immuno-suppressive and can be found in virtually all models of solid tumors and functionally similar cells may accumulate during autoimmune conditions and chronic inflammation (reviewed in [10]. In cancer, solid tumors produce various soluble factors such as VEGF, GM-CSF, M-CSF, IL-10 and TGFβ as well as certain inflammatory cytokines IL-6 and IL-1β that are involved in inducing the development of MDSC (reviewed in [6], [8], [9]). The suppressive Gr-1^+^ CD11b^+^ cell population has been sub-divided into two functional subsets, Ly6G^+^ CD11b^+^ granulocytic MDSC and Ly6C^hi^ CD11b^+^ monocytic MDSC, respectively [11], [12]. Thus, the granulocytic MDSC were shown to suppress the T cell response mainly through the production of reactive oxygen species (ROS), while the monocytic MDSCs exerted suppression through elevated activity of the iNOS and Arginase I enzymes. Other investigators have defined functional sub-populations of MDSC based on their surface expression level of the Gr-1 molecule [13], [14]. Also using this strategy, functionally similar granulocytic and monocytic sub-populations could be defined.

In addition to the immunosuppression mediated by MDSCs, the immunosuppressive cytokine TGFβ is overexpressed by tumors and has multiple functions in development of cancer (Reviewed in [15], [16]. Thus, it operates not only at the level of the cancer cell but also influences tumor stroma, including the attraction of MDSCs. TGFβ is also a potent regulator of the adaptive immune response and is involved both in effector T cell polarization and in the effector function of regulatory T cells [17]–[19]. Further, TGFβ signaling has been shown to block the effector T cell response to certain tumor cells [20], [21]. Thus, blockade of TGFβ activity modulates the suppressive milieu generated by the tumor such that the adaptive anti-tumor immune response mediated CD8 T cell anti-tumor responses will be unleashed [22]–[24].

The S100A9 protein belongs to the S100 protein family [25]. It is a calcium-binding protein that is expressed primarily in neutrophil granulocytes and in some monocyte subsets [26]. It has recently been shown that in the presence of zinc S100A9 undergoes a conformational change and becomes a ligand for the pro-inflammatory receptors Receptor for Advanced Glycation End products (RAGE) and Toll like receptor 4 (TLR4) [27], [28]. S100A9 is mostly expressed as a heterodimer together with S100A8, another member of the S100 protein family [26]. Elevated levels of these proteins have been observed in patients with inflammatory disease [29], but also in patients with prostate cancer [30]. S100A9 can also be expressed on the cell surface of immature monocytes [31], but its biological function as a cell surface protein is largely unknown. A S100A9-binding small molecule (ABR-215050) is presently in a Phase III clinical trial for the treatment of prostate cancer [32] [33].

S100A9 has been shown to be involved in the development of malignant disease. An increased expression of S100A9 can be detected in the pre-metastatic lung at sites where immature myeloid cells subsequently will be deposited [34]. Also, in colitis-induced colon cancer an increased expression of S100A8/A9 as well as RAGE has been reported [35]. Importantly, S100A9 expression has also been shown to be involved in MDSC function. Further, in the EL4 lymphoma model reduced tumor growth was observed in S100A9^−/−^ animals compared to wildtype controls [36]. This coincided with the reduction of the number of MDSC in S100A9^−/−^ animals. Also TLR4 has been shown to be involved in tumor progression [37], [38] in different tumor models. Thus, both S100A9 and its receptor TLR4 may be involved in tumor development.

We here report our findings concerning the role of S100A9 and TLR4 expression using the spontaneous prostate cancer model TRAMP, as well as the transplanted, syngeneic EL4 lymphoma model.

## Results

### TRAMP mice on S100A9^−/−^ genetic background have a delay in tumor growth

**Figure 1:**
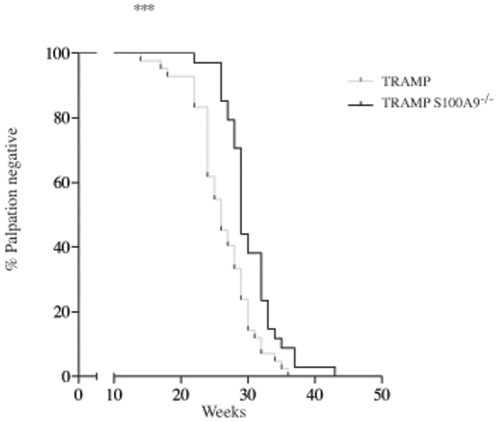
TRAMP tumor growth is delayed in S100A9^−/−^ mice. Time to palpable tumor curves for C57BL/6 TRAMP mice (grey line; n = 42) and TRAMP S100A9^−/−^ mice (black line; n = 34). The median time to palpable tumor (TM) was 26 and 29 weeks, respectively (p = 0.0008; Gehan-Breslow-Wilcoxon).

As mentioned above, S100A9 has been shown to be important for MDSC function and growth of the EL4 lymphoma in C57BL/6 mice [36]. We wanted to address whether S100A9 expression could influence the growth of a solid cancer in a spontaneous prostate cancer model [1]. To this end, we back-crossed S100A9^−/−^ mice for 10 generations onto C57BL/6 mice and subsequently crossed them with TRAMP mice on the same genetic background. The animals were followed by palpation once weekly from 10 weeks of age. Animals with a palpable tumor were sacrificed and the presence of a prostate tumor confirmed by necropsy. Figure 1 shows the time to palpable tumor for C57BL/6 TRAMP mice in grey (n = 42) and the TRAMP S100A9^−/−^ animals (n = 34) in black. The median time to palpable tumor (MT) was increased in TRAMP S100A9^−/−^ animals from 26 to 29 weeks (p = 0.0008; Gehan-Breslow-Wilcoxon test). We concluded from these data that the absence of S100A9 expression delays TRAMP tumor growth.

### S100A9 expression in TRAMP animals and human prostate cancer

**Figure 2:**
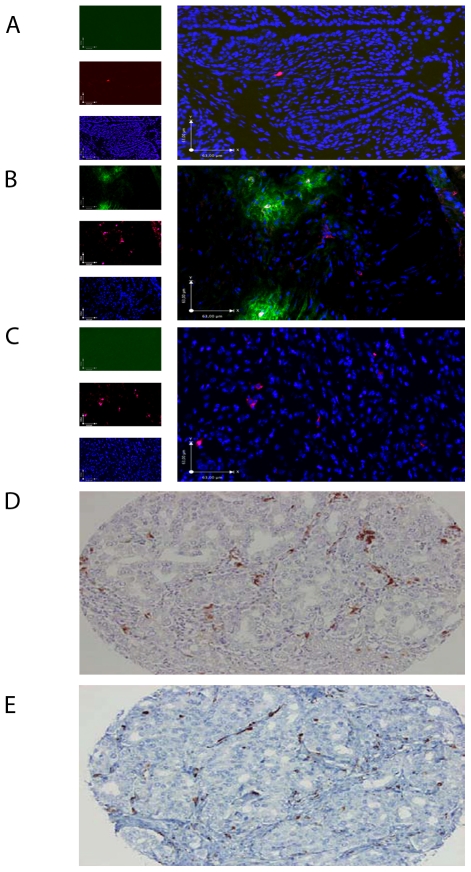
Monocytes/macrophages are the main S100A9 producing cells in TRAMP prostate and human prostate tumors. IHC analysis of prostate sections from C57BL/6 mice, TRAMP prostate tumors and TRAMP prostate tumors in S100A9^−/−^ mice (A–C). A representative section from one out of 5 analysed is shown. A. Left: Anti-S100A9 (green), anti-CD11b (purple) and Hoechst (blue) from C57BL/6 prostate tissue. Right: A merged image. Magnification ×20. B. Left: Anti-S100A9 (green), anti-CD11b (purple) and Hoechst (blue) from a TRAMP tumor in C57BL/6 mice. Right: A merged image. Magnification ×20. C. Left: Anti-S100A9 (green), anti-CD11b (purple) and Hoechst (blue) from a TRAMP tumor in C57BL/6 S100A9^−/−^ mice. Right: A merged image. Magnification ×20. Scale bars in panel A–C equals 63 mm. IHC analysis of human prostate cancer. Numerous S100A9 positive cells are observed in the tumor stroma. E. Consecutive section from the same tumor stained with an antibody against CD68 expressed on tumor infiltrating monocytes/macrophages.

Having established that S100A9 expression had an impact on tumor growth we proceeded to perform an immunoflourescence analysis of tumors from TRAMP mice. We first stained normal prostate from C57BL/6 animals and TRAMP tumors for S100A9 expression. As shown in Figure 2A, normal prostate tissue from C57BL/6 mice showed no S100A9 expression. In contrast, TRAMP tumors in C57BL/6 mice showed focal expression of S100A9 (Figure 2B). As a control, TRAMP tumors from S100A9^−/−^ animals showed no S100A9 expression (Figure 2C), indicating that our staining was specific and that tumors from TRAMP mice do not express S100A9. Lastly, co-staining with an anti-CD11b reagent revealed that in nearly all of the S100A9 expressing foci we could observe a centrally positioned, CD11b^+^ cell (Figure 2B). We conclude from these results that S100A9 production is induced in TRAMP tumors, and that the most likely primary source of the protein are CD11b^+^ cells and not tumor cells.

We next wanted to verify that S100A9 expression could also be detected in human prostate cancers. For this purpose we stained samples from benign and tumor tissue in a tissue micro array (TMA) containing 16 men with prostate cancer. As shown in Figure 2D, also in human prostate tumors there was S100A9 expression which coincided with infiltrating macrophage-like inflammatory cells in the tumor stroma. Occasionally focal staining was also observed in tumor epithelial cells (data not shown). The number of S100A9 stained inflammatory cells was higher in tumors than in the surrounding non-malignant prostate tissue. Some tumors contained many S100A9 positive immune cells whereas the number was low in others. There was no obvious relation between the number of S100A9 stained immune cells and tumor Gleason score. The number of S100A9 positive cells in the tumor stroma correlated to the number of tumor infiltrating CD68 positive macrophages (Figure 2E). We conclude from these preliminary data that S100A9 expression is up-regulated in prostate cancer tissue compared to adjacent normal prostate and that this expression seems to correlate with infiltrating macrophage-like cells.

### TRAMP mice on TLR4^−/−^ genetic background have a delay in tumor growth

**Figure 3:**
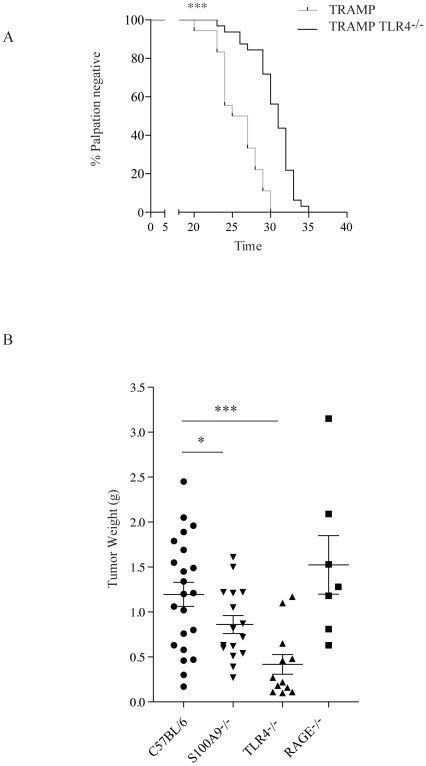
TRAMP tumor growth is delayed in TLR4^−/−^ mice and EL4 lymphoma growth in both S100A9^−/−^ and TLR4^−/−^ animals. A. Time to palpable tumor curves for C57BL/6 TRAMP mice (black line; n = 18) and TRAMP TLR4^−/−^ mice (dashed line; n = 32). The median time to palpable tumor (TM) was 26 and 31 weeks, respectively (p = <0.0001; Gehan-Breslow-Wilcoxon). B. Tumor weight of EL4 lymphoma tumors scored 14 days after subcutaneous inoculation in C57BL/6, S100A9^−/−^, RAGE^−/−^ and TLR4^−/−^ animals. Statistical analysis using two-tailed t test *** p = 0.0008; * p = 0.039.

S100A9 has been shown to be an endogenous TLR4 ligand [27]. Furthermore, TLR4 has been shown to be involved in tumor progression [37], [38], and we therefore wanted to investigate whether TLR4 expression could also influence tumor growth in the TRAMP tumor model. To this end, TRAMP animals were back-crossed with TLR4^−/−^ mice and monitored for progression to palpable tumor as above. The result showed that the MT in the control mice in this experiment was 26 weeks, while in TLR4^−/−^ mice MT was extended to 31 weeks (Figure 3A; p<0.0001).

Although the TRAMP model is an established and well-studied tumor model it had limitations for our continued studies. It being a spontaneous model makes it difficult to obtain age-matched animals to perform properly controlled experiments. Also, extraction of various tumor cell populations is, in our hands, very inefficient. It has been shown by others that tumor growth is delayed in S100A9^−/−^ mice also in the EL4 lymphoma model [36]. Therefore, we next investigated whether EL4 lymphoma growth was also influenced by TLR4 expression. As shown in Figure 3B, this was indeed the case. The tumor weight at 14 days after inoculation, was significantly reduced in TLR4^−/−^ animals. We also observed a significant reduction of tumor weight in S100A9^−/−^ animals, as previously published (17) (Figure 3B). Thus, we decided to continue our studies on the effect of S100A9 and TLR4 on tumor growth in the EL4 tumor system.

We then extended our studies using the EL4 lymphoma model to involve another receptor that has been shown to have S100A9 as a ligand; RAGE [27]. RAGE has primarily been studied as a pro-inflammatory receptor but has also been shown to be involved in tumor progression [39]. We therefore investigated whether growth of EL4 lymphoma cells were compromised in RAGE^−/−^ animals. Figure 3B shows that the EL4 lymphoma tumor growth was similar in RAGE^−/−^ animals compared to controls 14 days after inoculation. We conclude from these experiments that the inhibition of tumor growth observed in the S100A9^−/−^ mice might be dependent on interactions between S100A9 and TLR4 while RAGE may have no dominant role in this tumor model.

### CD11b^+^ cell subpopulations in S100A9^−/−^ and TLR4^−/−^ animals

**Figure 4:**
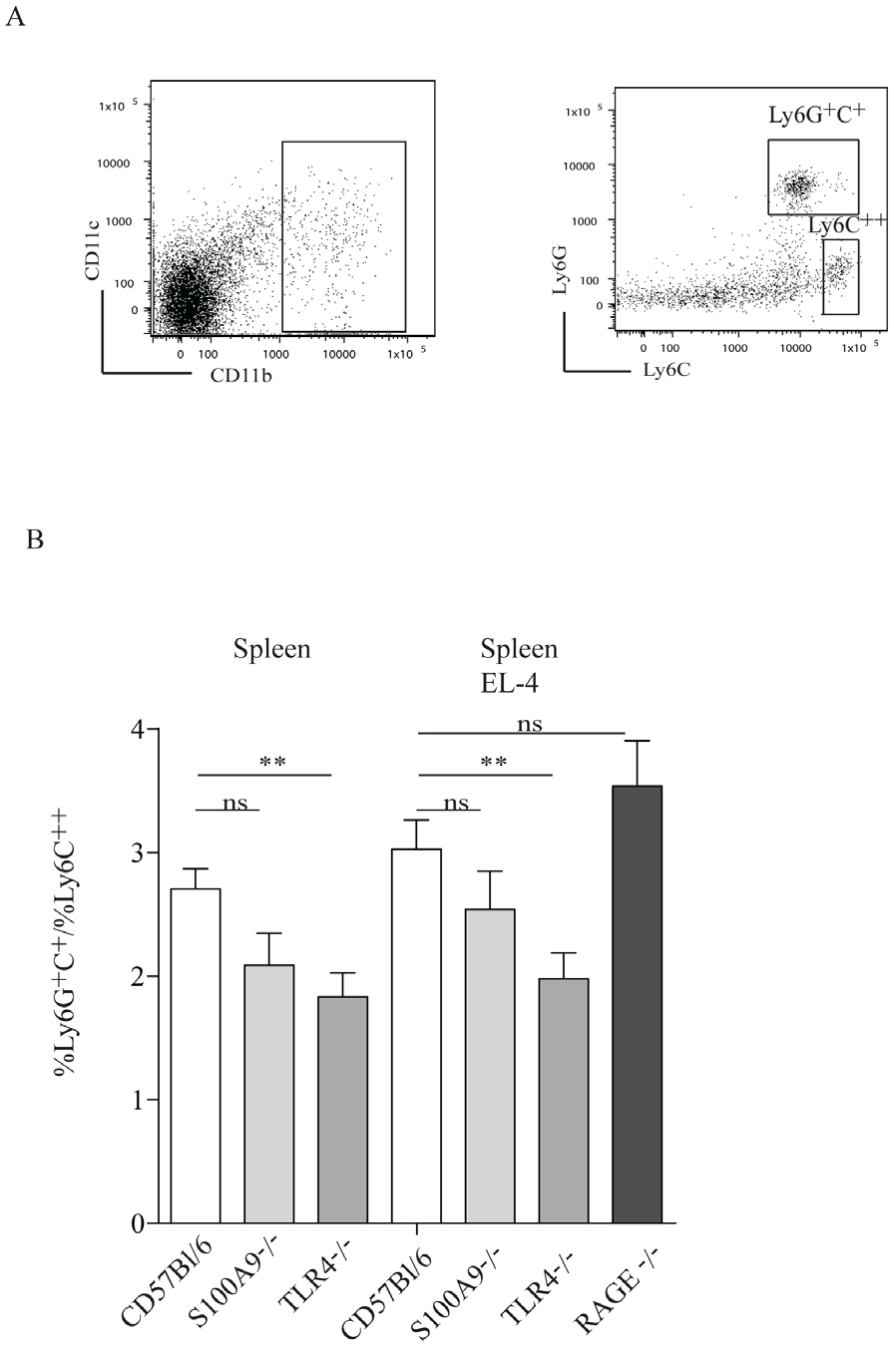
The CD11b^+^Ly6G^+^Ly6C^+^ cell population is reduced in spleens from S100A9^−/−^ and TLR4^−/−^ animals. FACS analysis of spleen cells from C57BL/6, S100A9^−/−^, TLR4^−/−^ and RAGE^−/−^ animals. Panel A: Left: The gate used for defining CD11b^+^ cells is shown. Right: The Ly6C/Ly6G populations used for comparison and defined as indicated. Panel B: The ratio of CD11b^+^Ly6G^+^C^+^/CD11b^+^Ly6C^++^ cells in the different mouse strains in the presence and absence of subcutaneous EL4 tumor, as indicated. Statistical analysis using two-tailed t test ** p = 0.0034.

S100A9 has been shown to be expressed in immature myeloid cells [31] and this cellular compartment has been shown to be functionally compromised in S100A9^−/−^ animals [36]. We therefore investigated whether the absence of S100A9 and TLR4 expression had any impact on the CD11b^+^ cell subpopulations. To this end, age matched C57BL/6 mice, TLR4^−/−^, S100A9^−/−^ and RAGE^−/−^ animals were inoculated with EL4 cells subcutaneously. Two weeks later the CD11b^+^ cells in spleen were stained for their Ly6C and Ly6G expression and analyzed by FACS (Figure 4A). The ratio of CD11b^+^Ly6G^+^Ly6C^+^/CD11b^+^Ly6C^++^ cells was significantly reduced in TLR4^−/−^ animals compared to C57BL/6 control animals (Figure 4B). In animals inoculated with EL4 tumors the CD11b^+^Ly6G^+^Ly6C^+^/CD11b^+^Ly6C^++^ ratio was increased in all three mouse strains, but was still significantly lower in TLR4^−/−^ animals than in C57BL/6 controls (Figure 4B). We have also made the analysis looking at absolute cell numbers rather than percentages which revealed that the CD11b ratio of Ly6G^+^ granulocytic population is reduced relative to the Ly6C^+^ monocytic population, without significantly reducing the total number of CD11b^+^ cells (data not shown). We therefore conclude from these experiments that the relative composition of splenic CD11b^+^ cells with regard to the Ly6G and Ly6C markers is altered in mice lacking either S100A9 or TLR4 without changing the total number of CD11b^+^ cells.

### TGFβ expression is down-regulated in S100A9^−/−^ and TLR4^−/−^ animals

**Figure 5:**
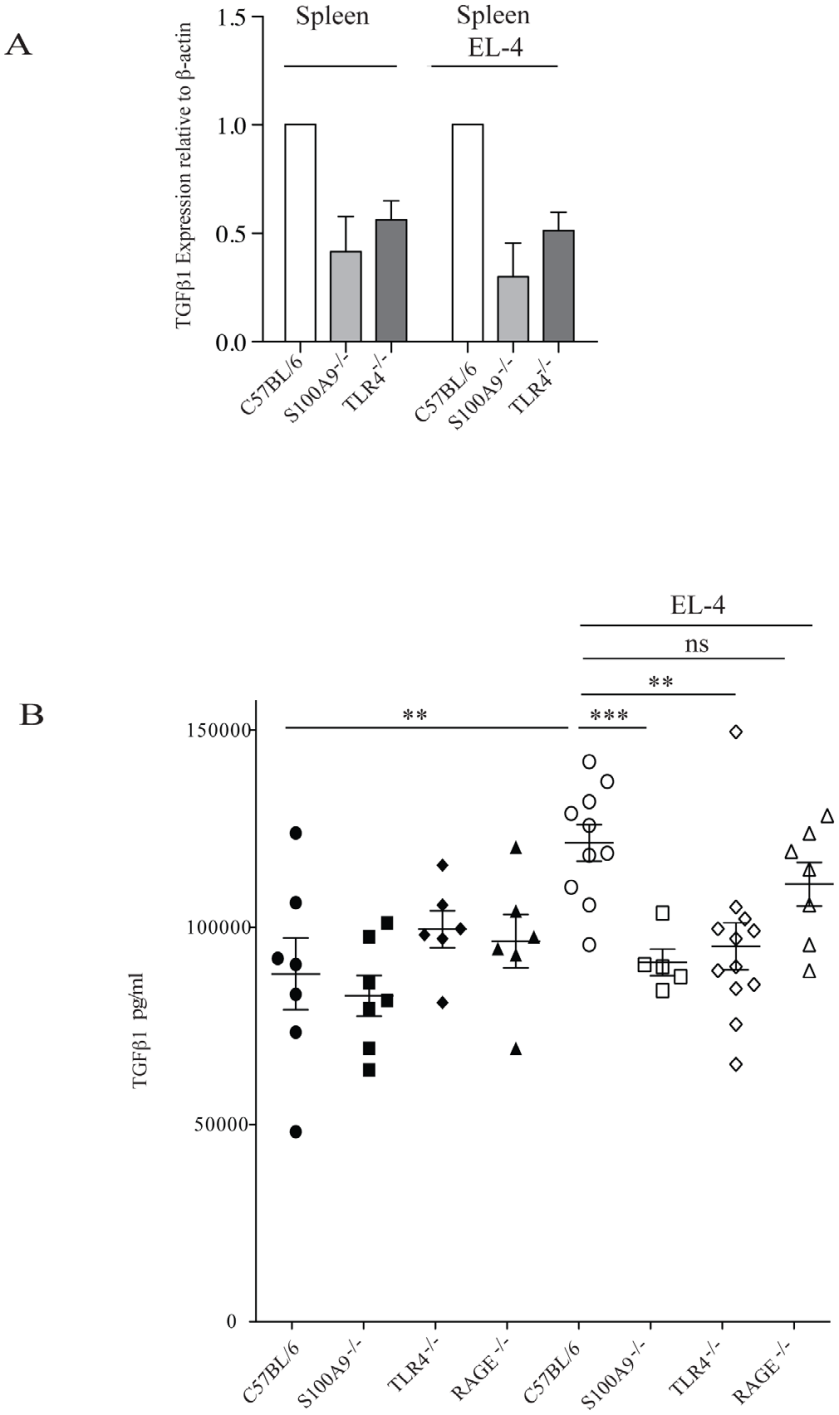
TGFβ expression is reduced in S100A9^−/−^ and TLR4^−/−^ animals. A. Quantitative real time RT-PCR analysis (see Materials and Methods) of TGFβ RNA expression from CD11b^+^ cells (>90% pure by FACS analysis) from the spleen of C57BL/6, S100A9^−/−^ and TLR4^−/−^ animals in the absence of, or 14 days after inoculation, with 50,000 EL4 lymphoma cells subcutaneously. The mean expression from 4 separate experiments is shown where the expression in the C57BL/6 controls have been set to 1. B. ELISA measurements of TGFβ serum levels of C57BL/6 (filled circles), RAGE^−/−^ (filled triangles), S100A9^−/−^ (filled squares) and TLR4^−/−^ (filled diamonds) in the absence of, or 14 days after inoculation (open symbols), with 50,000 EL4 lymphoma cells subcutaneously. Statistical analysis using two-tailed t test *** p = 0.0008; * p = 0.039 and 0.0028, respectively. There was no statistical significant difference in TGFβ serum levels between EL4 inoculated C57BL/6 or RAGE^−/−^ mice.

We next investigated the expression of immune regulating genes by RT-PCR in CD11b^+^ cells from the spleen of animals with and without EL4 tumors. In addition, we performed this experiment in parallel in C57BL/6, TLR4^−/−^ and S100A9^−/−^ animals. The rational for this approach was that critical mediators should show the same deviation in TLR4^−/−^ and S100A9^−/−^ animals compared to C57BL/6 controls if they could be involved in the observed *in vivo* effect on tumor growth regulation. When the RNA expression of arginase I, IFNγ, RAGE and iNOS were investigated no such co-variation was observed (Figure S1). However, TGFβ RNA expression was significantly higher in splenic CD11b^+^ cells from naive C57BL/6 animals compared to S100A9^−/−^ and TLR4^−/−^ mice. This difference was found in control as well as in EL4 tumor bearing mice (Figure 5A).

To validate the observed effect on TGFβ RNA expression we next measured TGFβ protein levels in serum from C57BL/6, TLR4^−/−^, S100A9^−/−^ and RAGE^−/−^ animals. As shown in Figure 5B, the serum level in healthy animals were similar in all four mouse strains. After inoculation with EL4 lymphoma cells the expression level of TGFβ was increased in C57BL/6 and RAGE^−/−^, but not in S100A9^−/−^ or TLR4^−/−^ animals. Thus, the reduced tumor growth observed in S100A9^−/−^ and TLR4^−/−^ animals correlate with low TGFβ RNA expression in splenic CD11b^+^ cells. Furthermore, TGFβ systemic protein expression is not induced after inoculation with tumor cells in these mouse strains compared to controls. EL4 lymphoma cells expressed only low levels of TGFβ RNA both *in vitro* and was not in our hands induced *in vivo* (Figure S2).

### Interruption of S100A9/TLR4 interaction in vivo inhibits EL4 cell growth

**Figure 6:**
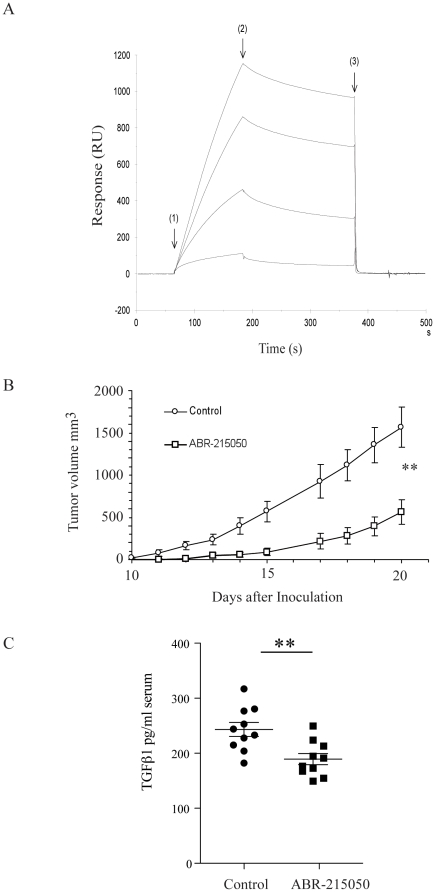
An S100A9-binding small molecule inhibits EL4 lymphoma growth *in vivo*. A. Binding of S100A9 to immobilized TLR4/MD2 complex is blocked by ABR-215050. Sensorgrams obtained after injection (2 min at 30 µL/min) of 50 nM S100A9 ± ABR-215050 over amine coupled TLR4/MD2 (density ∼2.3 kRU). Sensorgrams from top to bottom: S100A9 without competitor and with 3.91, 31.25 and 1,000 µM ABR-215050. Arrows indicate injection of sample (1); sample buffer - i.e. HBS-P containing 1 mM Ca^2+^ and 10 uM Zn^2+^ (2); and regeneration of surface with 3 M EDTA (3). B. Anti-tumor effect of ABR-215050 in EL4 tumors inoculated (s.c.) into wild type mice. The ABR-215050 was administrated in the drinking water at 30 mg/kg/day seven days/week from day 0 throughout the experiment. Each data point represents mean ± SEM (n = 10; p<0.01, Mann Whitney U). Control animals received only normal drinking water. The water intake of the animals was not affected by the presence of ABR-215050 in the drinking water. C. ELISA measurements of TGFβ serum levels day 20 in C57BL/6 animals inoculated with EL4 tumors and animals treated with 30 mg/kg/day of ABR-215050, as indicated (p = 0.0037, Student t test).

It has been shown that compounds belonging to the quinoline-3-carboxamide class will bind to S100A9 and inhibit the interaction between S100A9 and TLR4 or RAGE [27]. One compound (ABR-215050; tasquinimod) has entered phase III clinical testing for the treatment of prostate cancer and has been shown to have anti-tumor activity in animal tumor models [40], [41]. The ABR-215050 compound inhibits in a dose dependent way the interaction between S100A9 and TLR4/MD2 (Figure 6A) or RAGE (Figure S3 [27]). To directly test whether the S100A9/TLR4/RAGE interaction was important for EL4 tumor growth *in vivo* we therefore treated EL4 inoculated animals with 30 mg/kg ABR-215050 and compared to control animals. As shown in Figure 6B, this treatment significantly reduced EL4 tumor growth. In addition, when the experiment was repeated and the serum levels of TGFβ in serum was analysed in control and ABR-215050 treated animals, a significant reduction of TGFβ expression upon treatment was observed (Figure 6C). Taken together with the results above from S100A9^−/−^, TLR4^−/−^ and RAGE^−/−^ animals we conclude that a molecular interaction between S100A9 and TLR4 appears to be stimulatory for tumor cell growth in this experimental system, and that TGFβ expression may be a surrogate marker for S100A9/TLR4 interaction *in vivo*.

## Discussion

We have shown here that the absence of S100A9 or TLR4 expression delays tumor incidence in a spontaneous prostate cancer model. After the initiation of the current investigation, Cheng *et al.* [36] published an elegant study where they showed that S100A9^−/−^ animals were protected from growth of transplantable tumors (EL4 lymphoma). They also showed that MDSC had an increased expression of the S100A9 protein. Furthermore, over-expression of S100A9 or S100A8/A9, but interestingly not S100A8, promoted tumor growth and MDSC development in parallel. The difference between the TRAMP model studied here and a transplantable tumor is that the tumors develop during a long time, typically >20 weeks, in a fully immune competent host. This means that the immune system has ample time to adapt to the signals released by a tumor. Thus, in the present investigation, in contrast to that of Cheng *et al.* [36], all the TRAMP/S100A9^−/−^ animals developed palpable tumors, although significantly later than the C57BL/6 control animals. The same observation was true for TRAMP/TLR4^−/−^ mice while in contrast many TLR4^−/−^ animals did not develop EL4 tumors. This observation could be explained by that although S100A9^−/−^, and possibly also TLR4^−/−^ animals, may have a compromised MDSC function, sufficient numbers of these cells will with time accumulate and be able to down-regulate the immune surveillance function of the host in the TRAMP model.

We also show here that TLR4 expression, like S100A9 expression [36], influences the growth of the EL4 lymphomas. Hence, the effect of S100A9 and TLR4 on the growth of TRAMP prostate tumors is also reflected in the growth of EL4 lymphoma. Interestingly, while S100A9 has been shown to be a ligand both for RAGE and TLR4 [27], no effect on EL4 lymphoma growth was observed in RAGE^−/−^ mice. Therefore, we suggest that it is primarily the interaction between S100A9 and TLR4 that promotes tumor growth in our studies.

To further strengthen that statement we also show that a small molecule, ABR-215050, that inhibits the interaction between S100A9 and TLR4 also inhibits EL4 lymphoma growth. ABR-215050 has been shown to inhibit tumor growth in several models of prostate cancer [40], partially explained by an anti-angiogenic effect [41]. This molecule has been in clinical testing and shown positive results in phase I and phase II clinical trials for the treatment of castrate resistant, metastatic prostate cancer [32], [33]. It is currently in phase III clinical development for the same indication (see www.clinicaltrials.gov). Hence, our observations in this experimental system might have a corollary in human disease which would extend the implications of the role of S100A9 interactions in human disease.

S100A9 in tumors appeared to be primarily expressed by CD11b^+^ cells. In sections from human prostate cancer biopsies, many immune cells in the stroma could be seen to express S100A9 and the number of CD68^+^ macrophages correlated with the number of S100A9^+^ foci. Also, compared to normal prostate in mice or adjacent normal prostate tissue in human biopsies, S100A9 expression was found mostly in tumor tissue. With regard to TLR4 we have been unable to detect any expression using immuno flourescence, while we have observed occasional RAGE expression in TRAMP tumors (data not shown). However, when EL4 lymphoma growth was investigated using RAGE^−/−^ mice we could not detect any reduction of tumor cell growth. Since RAGE has been shown to be important in tumor growth in other systems [39], it is not far fetched to suggest that the effect of the S100A9/RAGE interaction might be different than the S100A9/TLR4 interaction in the regulation of tumor growth.

Concerning the effects on MDSC we could detect that C57BL/6 mice inoculated with EL4 tumors showed an increased ratio between CD11b^+^Ly6C^+^G^+^/Ly6C^++^ cells in the spleen. In fact this dominance in CD11b^+^Ly6C^+^G^+^ MDSC was detected in several different tumor models [12]. Interestingly, this ratio was lower in S100A9^−/−^ and TLR4^−/−^ mice. In animals inoculated with EL4 tumors the CD11b^+^Ly6C^+^G^+^/Ly6C^++^ ratio was increased in all mouse strains but remained lower in S100A9^−/−^ and TLR4^−/−^ mice. In RAGE^−/−^ animals inoculated with EL4 tumors the CD11b^+^Ly6C^+^G^+^/Ly6C^++^ ratio was even higher than in C57BL/6 controls. Hence, the mouse strains that show a reduced growth of EL4 lymphoma cells have a lower ratio of CD11b^+^Ly6C^+^G^+^/Ly6C^++^ cells in the spleen. We therefore believe that the CD11b^+^Ly6C^+^G^+^ MDSC may be more important in promoting tumor growth in this model. Interestingly, CD11b^+^Ly6G^+^ cells is the subpopulation of CD11b^+^ cells that express the highest level of S100A9 (Figure S4). Thus, it can be speculated that these cells release S100A9 that via interaction with TLR4 promote tumor growth. Lastly, these data also indicate that S100A9, RAGE, or TLR4 expression does not appear to be absolutely required for the expansion of CD11b^+^ cells.

When splenic CD11b^+^ cells were analyzed for the expression of immunomodulatory genes using RT-PCR, the only significant difference detected in both S100A9^−/−^ and TLR4^−/−^ animals compared to C57BL/6 controls, both in animals with or without EL4 tumors, was TGFβ RNA expression. When this analysis was extended to TGFβ expression at the protein level we found that while TGFβ serum levels in C57BL/6 and RAGE^−/−^ mice increased in tumor-inoculated animals it did not in S100A9^−/−^ and TLR4^−/−^ animals. However, unlike the RNA data TGFβ systemic levels were not reduced in S100A9^−/−^ and TLR4^−/−^ animals indicating that other cells than splenic CD11b^+^ cells contribute to the systemic level of TGFβ. Also, when tumor inoculated animals were treated with ABR-215050 the reduction of tumor growth coincided with reduced serum TGFβ levels. This is most likely not due to the relative lower tumor burden since the EL4 tumor used expressed only very low levels of TGFβ both *in vitro* and *in vivo* (Figure S2). TGFβ plays multiple roles in cancer and is also involved in regulating the adaptive immune response both to tumors and infection (reviewed in [15]–[17], [19]. We therefore believe that the correlation between TGFβ expression and tumor development described in here may be of significance. Whether the reduced production of TGFβ mediates the observed reduction in tumor growth, and whether the skewed ratio of CD11b^+^Ly6C^+^G^+^/Ly6C^++^ cells is involved in the control of TGFβ levels, remains to be determined.

In conclusion, besides its involvement in the metastatic process [34], S100A9 also appears to be involved in the growth control of established tumors. The interference with S100A9 signaling may therefore present a new treatment modality for managing malignant disease, for example in the prostate. Particularly as human prostate tumors contain numerous S100A9 expressing inflammatory cells and also some S100A9 expressing tumor epithelial cells [30], and the overall effect of infiltrating monocytes is to stimulate prostate tumor growth [42], [43]. The result from ongoing clinical trials with S100A9-binding compounds will add further information concerning this possibility.

## Materials and Methods

### Animals

C57BL/6 mice, S100A9^−/−^ [44], TLR4^−/−^, RAGE^−/−^ and TRAMP [1] (Jackson Laboratories) mice were kept in an SPF animal facility at BMC, Lund. Female C57Bl/6 TRAMP mice heterozygous for the Probasin SV-40 Tag transgene were bred to non-transgenic C57Bl/6 males (Taconic M&B, Ry, Denmark), as well as to TLR4^−/−^ and S100A9^−/−^ on a C57BL/6 background. Ear DNA was isolated from all mice, and transgenic animals were identified via PCR based screening after each crossing. TRAMP tumors were scored by blinded palpation and sacrificed upon being palpation positive, according to local ethical guidelines. Necropsy was performed on all animals and only animals that had a macroscopically visible prostate tumor were scored as tumor bearing in the data set presented.

### Human prostate cancer samples

To stain human prostate tissue for S100A9 and CD68 (monocytes) as described below, we used tissue micro arrays (TMA) constructed from paraffin blocks from 403 prostate cancer patients as described earlier [45]. Each patient was represented by up to 8 cores of tumor and up to 4 cores of adjacent non-malignant tissue. TMA cores from 16 patients were stained and analyzed.

### Immunohistochemistry

Tissues analysed with immunohistology were embedded in OCT compound (Tissue-Tek®), and snap-frozen in liquid nitrogen. Cryosections (5–6 µm) were prepared on microscope slides, air dried and frozen at −20°C until staining procedures. PFA fixed sections were incubated with blocking 1% BSA 10% serum and FcRII/III blocker solution followed by Avidin/Biotin Blocking kit (Vector Laboratories, Inc. Burlingame, CA, USA). Thereafter the sections were incubated for 30 min at room temperature with primary antibodies: Rabbit-anti-S100A9, or the appropriate isotype controls (BD Pharmingen). Followed by Donkey- anti- rabbit Alexa488 (Molecular Probes) and anti-mouse CD11b-APC conjugate (eBioscience San Diego CA, USA). The slides were mounted using ProLong Gold mounting media (Invitrogen, Oregon, USA). Images were acquired with a Axiovert 200 M Zeiss microscope (Carl Zeiss, Inc.) connected to a ORCA-ER CCD camera (Hamamatsu, Japan), and analysed with Volocity software (Improvision), or AxioVision 4.0 software (Carl Zeiss, Inc.).

Five micron thick paraffin sections from the TMAs were stained for CD68 using an antibody from DAKO, Stockholm, Sweden and for S100A9 using an antibody from Santa Cruz Biotechnology, CA, USA as earlier described [42], [46].

### Flow cytometry

Flow cytometric analysis was performed on spleen cell suspensions, as indicated. Primary Abs used were: anti-mouse CD11b APC (eBioscience), Ly6G-FITC (BD Pharmingen) and Ly6C biotin (BD Pharmingen). Biotinylated antibodies were detected with streptavidin-QD605 (Invitrogen). Propidium iodide was added to samples before acquisition. Data were acquired using a FACSAria flow cytometer (BD Biosciences) and analyzed using FlowJo software (Tree Star).

### Surface Plasmon Resonance (SPR) analysis

Analysis of S100A9 binding to TLR4 was carried out on a BIAcore 3000 System (GE Healthcare). Recombinant TLR4/MD2 complex was immobilized on a CM5 sensor chip (GE Healthcare) to a density of ∼2,300 RU by coupling via primary amines. S100A9, in the absence or presence of ABR-215050, was injected (2 min at 30 µL/min) at 1.3 µg/mL (50 nM homodimeric concentration) in HBS-P buffer (10 mM Hepes, 0.15 M NaCl, 0.005% v/v Surfactant P20, pH 7.4) containing 1 mM Ca^2+^ and 10 µM Zn^2+^. Regeneration of the surface between each cycle was performed by injection of 30 µL HBS-P containing 3 M EDTA.

### EL4 lymphoma model and RT-PCR

C57BL/6, S100A9^−/−^, TLR4^−/−^ and RAGE^−/−^ mice (12 weeks old) were injected subcutaneously with 50,000 EL4 lymphoma cells in 100 µl PBS. As control, 100 µL PBS alone were injected. After 14 days the animals were scored for tumor growth by palpation. All tumor positive animals were verified for tumor growth by autopsy. In some experiments the tumors where extracted and quantitated by weighting, while in others tumor growth was measured with a microcaliper and tumor volume was calculated every day, starting day 10 throughout the experiment, as indicated. The tumor volume was calculated as volume = L×W×W×0.4, where L is the length (mm) and W (mm) is the width of the tumor 6 [47].

Spleens were dissected and the cell suspension was thereafter passed through a 70 µm cell strainer and cells washed in Hank's BSS (Invitrogen Life Technologies, Paisley, UK). Splenic CD11b^+^ cells were purified using anti-CD11b magnetic beads and LS-columns (Miltenyi Biotech, Bergisch Gladbach, Germany). Total RNA was extracted from CD11b^+^ cell preparations by use of the Purelink RNA mini Kit (Invitrogen). RNA was reverse transcribed to cDNA by use of the SuperScript III Platinum synthesis system (Invitrogen). Real-time PCR (RT-PCR) was performed for the detection of TGFβ RNA and quantified using a SYBR GreenER kit (Invitrogen) in a MYIQ (Bio-Rad) PCR machine. The threshold cycle number was determined and relative expression level of each mRNA was determined using the formula 2^(Rt– Et)^, where Rt and Et are the threshold cycles for the reference gene (b-actin) and the target gene, respectively. Where mean values from several experiments are shown the expression in C57BL/6 controls have been set to 1.

### TGFβ ELISA

The TGFβ1 Quantikine ELISA kit (Abnova Taipei, Taiwan) was utilized to determine serum TGFβ levels, as indicated. Serum from individual animals in normal and EL-4 tumor bearing animals and acid activated as per manufacturers direction. Samples were diluted and assayed in as described by the manufacturer. Prism 4 software was used to construct a TGFβ standard curve and subsequently to quantitate sample TGFβ concentrations. Cumulative TGFβ levels over all time points were graphed using Prism 4.

### Statistical analysis

Statistical analysis was performed with Prism 4 software (GraphPad Software, Inc.) using an unpaired two-tailed Student's *t* test or Gehan-Breslow-Wilcoxon test, as indicated. Error bars represent SEM. Statistical differences for the mean values are indicated as follows: *, P<0.05; **, P<0.01; ***, P<0.001.

## Supporting Information

Figure S1 RT-PCR analysis of the indicated genes using RNA from CD11b+ spleen cells, as described in Materials and Methods.
(TIF)

Figure S2 RNA from EL4 cells were analyzed with regard to TGFβ expression. CD3 expressing (MACS purification) cells from collagenase treated tumors were used as a source for EL4 *in vivo* RNA.
(TIF)

Figure S3 Upper panel: Recombinant S100A9 bind to amine coupled ABR-215050 in the presence of 1 mM Ca^++^ and 10 mM Zn^++^. Lower panel: ABR215050 inhibits the interaction between S100A9 and RAGE. (curve was fit to a sigmoidal dose-response model in GraphPad Prism).
(TIF)

Figure S4 Intra-cellular staining of S100A9 in spleens from C57BL/6 and S100^−/−^ mice. An affinity, purified rabbit anti-mouse S100A9 antibody was used to detect S100A9 in permeabilized cells.
(TIF)

## References

[1] Greenberg NM, DeMayo F, Finegold MJ, Medina D, Tilley WD (1995). Prostate cancer in a transgenic mouse.. Proc Natl Acad Sci U S A.

[2] Martiniello-Wilks R, Dane A, Mortensen E, Jeyakumar G, Wang XY (2003). Application of the transgenic adenocarcinoma mouse prostate (TRAMP) model for pre-clinical therapeutic studies.. Anticancer Res.

[3] Wikstrom P, Lindahl C, Bergh A (2005). Characterization of the autochthonous transgenic adenocarcinoma of the mouse prostate (TRAMP) as a model to study effects of castration therapy.. Prostate.

[4] Degl'Innocenti E, Grioni M, Boni A, Camporeale A, Bertilaccio MT (2005). Peripheral T cell tolerance occurs early during spontaneous prostate cancer development and can be rescued by dendritic cell immunization.. Eur J Immunol.

[5] Peranzoni E, Zilio S, Marigo I, Dolcetti L, Zanovello P (2010). Myeloid-derived suppressor cell heterogeneity and subset definition.. Curr Opin Immunol.

[6] Ribechini E, Greifenberg V, Sandwick S, Lutz MB (2010). Subsets, expansion and activation of myeloid-derived suppressor cells.. Med Microbiol Immunol.

[7] Youn JI, Gabrilovich DI (2010). The biology of myeloid-derived suppressor cells: the blessing and the curse of morphological and functional heterogeneity.. Eur J Immunol.

[8] Gabrilovich DI, Nagaraj S (2009). Myeloid-derived suppressor cells as regulators of the immune system.. Nat Rev Immunol.

[9] Marigo I, Dolcetti L, Serafini P, Zanovello P, Bronte V (2008). Tumor-induced tolerance and immune suppression by myeloid derived suppressor cells.. Immunol Rev.

[10] Ostrand-Rosenberg S, Sinha P (2009). Myeloid-derived suppressor cells: linking inflammation and cancer.. J Immunol.

[11] Movahedi K, Guilliams M, Van den Bossche J, Van den Bergh R, Gysemans C (2008). Identification of discrete tumor-induced myeloid-derived suppressor cell subpopulations with distinct T cell-suppressive activity.. Blood.

[12] Youn JI, Nagaraj S, Collazo M, Gabrilovich DI (2008). Subsets of myeloid-derived suppressor cells in tumor-bearing mice.. J Immunol.

[13] Greifenberg V, Ribechini E, Rossner S, Lutz MB (2009). Myeloid-derived suppressor cell activation by combined LPS and IFN-gamma treatment impairs DC development.. Eur J Immunol.

[14] Dolcetti L, Peranzoni E, Ugel S, Marigo I, Fernandez Gomez A (2010). Hierarchy of immunosuppressive strength among myeloid-derived suppressor cell subsets is determined by GM-CSF.. Eur J Immunol.

[15] Massague J (2008). TGFbeta in Cancer.. Cell.

[16] Bierie B, Moses HL (2010). Transforming growth factor beta (TGF-beta) and inflammation in cancer.. Cytokine Growth Factor Rev.

[17] Li MO, Wan YY, Sanjabi S, Robertson AK, Flavell RA (2006). Transforming growth factor-beta regulation of immune responses.. Annu Rev Immunol.

[18] Rubtsov YP, Rudensky AY (2007). TGFbeta signalling in control of T-cell-mediated self-reactivity.. Nat Rev Immunol.

[19] Flavell RA, Sanjabi S, Wrzesinski SH, Licona-Limon P (2010). The polarization of immune cells in the tumour environment by TGFbeta.. Nat Rev Immunol.

[20] Gorelik L, Flavell RA (2001). Immune-mediated eradication of tumors through the blockade of transforming growth factor-beta signaling in T cells.. Nat Med.

[21] Thomas DA, Massague J (2005). TGF-beta directly targets cytotoxic T cell functions during tumor evasion of immune surveillance.. Cancer Cell.

[22] Ge R, Rajeev V, Ray P, Lattime E, Rittling S (2006). Inhibition of growth and metastasis of mouse mammary carcinoma by selective inhibitor of transforming growth factor-beta type I receptor kinase in vivo.. Clin Cancer Res.

[23] Suzuki E, Kim S, Cheung HK, Corbley MJ, Zhang X (2007). A novel small-molecule inhibitor of transforming growth factor beta type I receptor kinase (SM16) inhibits murine mesothelioma tumor growth in vivo and prevents tumor recurrence after surgical resection.. Cancer Res.

[24] Nam JS, Terabe M, Mamura M, Kang MJ, Chae H (2008). An anti-transforming growth factor beta antibody suppresses metastasis via cooperative effects on multiple cell compartments.. Cancer Res.

[25] Marenholz I, Heizmann CW, Fritz G (2004). S100 proteins in mouse and man: from evolution to function and pathology (including an update of the nomenclature).. Biochem Biophys Res Commun.

[26] Roth J, Vogl T, Sorg C, Sunderkotter C (2003). Phagocyte-specific S100 proteins: a novel group of proinflammatory molecules.. Trends Immunol.

[27] Bjork P, Bjork A, Vogl T, Stenstrom M, Liberg D (2009). Identification of Human S100A9 as a novel target for treatment of autoimmune disease via binding to quinoline-3-carboxamides.. PLoS Biology.

[28] Vogl T, Tenbrock K, Ludwig S, Leukert N, Ehrhardt C (2007). Mrp8 and Mrp14 are endogenous activators of Toll-like receptor 4, promoting lethal, endotoxin-induced shock.. Nat Med.

[29] Foell D, Roth J (2004). Proinflammatory S100 proteins in arthritis and autoimmune disease.. Arthritis Rheum.

[30] Hermani A, Hess J, De Servi B, Medunjanin S, Grobholz R (2005). Calcium-binding proteins S100A8 and S100A9 as novel diagnostic markers in human prostate cancer.. Clin Cancer Res.

[31] Foell D, Wittkowski H, Vogl T, Roth J (2007). S100 proteins expressed in phagocytes: a novel group of damage-associated molecular pattern molecules.. J Leukoc Biol.

[32] Bratt O, Haggman M, Ahlgren G, Nordle O, Bjork A (2009). Open-label, clinical phase I studies of tasquinimod in patients with castration-resistant prostate cancer.. Br J Cancer.

[33] Pili R, Häggman M, Stadler WM, Gingrich J, Assikis V, Björk A, Nordle Ö, Forsberg G, Carducci MA, Armstrong AJ (2011). Phase II Randomized Double Blind Placebo-Controlled Study to Determine the Efficacy of Tasquinimod in Asymptomatic Patients with Metastatic Castrate-Resistant Prostate Cancer.. J Clin Oncol.

[34] Hiratsuka S, Watanabe A, Aburatani H, Maru Y (2006). Tumour-mediated upregulation of chemoattractants and recruitment of myeloid cells predetermines lung metastasis.. Nat Cell Biol.

[35] Turovskaya O, Foell D, Sinha P, Vogl T, Newlin R (2008). RAGE, carboxylated glycans and S100A8/A9 play essential roles in colitis-associated carcinogenesis.. Carcinogenesis.

[36] Cheng P, Corzo CA, Luetteke N, Yu B, Nagaraj S (2008). Inhibition of dendritic cell differentiation and accumulation of myeloid-derived suppressor cells in cancer is regulated by S100A9 protein.. J Exp Med.

[37] Chen R, Alvero AB, Silasi DA, Steffensen KD, Mor G (2008). Cancers take their Toll–the function and regulation of Toll-like receptors in cancer cells.. Oncogene.

[38] Wang EL, Qian ZR, Nakasono M, Tanahashi T, Yoshimoto K (2010). High expression of Toll-like receptor 4/myeloid differentiation factor 88 signals correlates with poor prognosis in colorectal cancer.. Br J Cancer.

[39] Gebhardt C, Riehl A, Durchdewald M, Nemeth J, Furstenberger G (2008). RAGE signaling sustains inflammation and promotes tumor development.. J Exp Med.

[40] Isaacs JT, Pili R, Qian DZ, Dalrymple SL, Garrison JB (2006). Identification of ABR-215050 as lead second generation quinoline-3-carboxamide anti-angiogenic agent for the treatment of prostate cancer.. Prostate.

[41] Olsson A, Bjork A, Vallon-Christersson J, Isaacs JT, Leanderson T (2010). Tasquinimod (ABR-215050), a quinoline-3-carboxamide anti-angiogenic agent, modulates the expression of thrombospondin-1 in human prostate tumors.. Mol Cancer.

[42] Lissbrant IF, Stattin P, Wikstrom P, Damber JE, Egevad L (2000). Tumor associated macrophages in human prostate cancer: relation to clinicopathological variables and survival.. Int J Oncol.

[43] Halin S, Rudolfsson SH, Van Rooijen N, Bergh A (2009). Extratumoral macrophages promote tumor and vascular growth in an orthotopic rat prostate tumor model.. Neoplasia.

[44] Manitz MP, Horst B, Seeliger S, Strey A, Skryabin BV (2003). Loss of S100A9 (MRP14) results in reduced interleukin-8-induced CD11b surface expression, a polarized microfilament system, and diminished responsiveness to chemoattractants in vitro.. Mol Cell Biol.

[45] Chung SC, Hammarsten P, Josefsson A, Stattin P, Granfors T (2009). A high cannabinoid CB(1) receptor immunoreactivity is associated with disease severity and outcome in prostate cancer.. Eur J Cancer.

[46] Yanamandra K, Alexeyev O, Zamotin V, Srivastava V, Shchukarev A (2009). Amyloid formation by the pro-inflammatory S100A8/A9 proteins in the ageing prostate.. PLoS One.

[47] Attia MA, Weiss DW (1966). Immunology of spontaneous mammary carcinomas in mice. V. Acquired tumor resistance and enhancement in strain A mice infected with mammary tumor virus.. Cancer Res.

