# Supplementary figures and images for: S100A9 Interaction with TLR4 Promotes Tumor Growth
Source: PLoS One. 2012 Mar 28;7(3):e34207. doi: 10.1371/journal.pone.0034207 (PMC3314596; doi:10.1371/journal.pone.0034207)

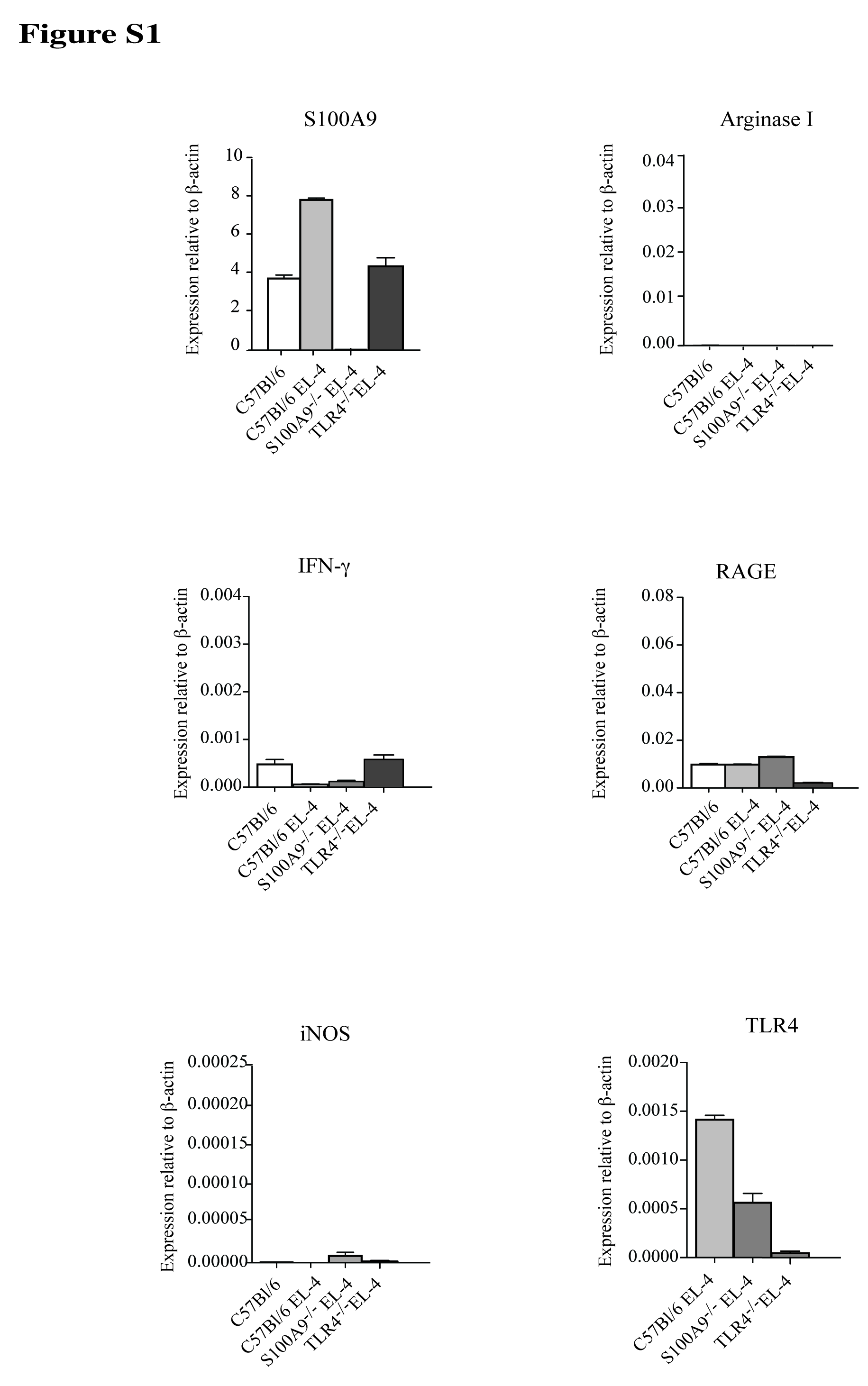

Supplement: Figure S1 — RT-PCR analysis of the indicated genes using RNA from CD11b+ spleen cells, as described in Materials and Methods. (TIF) [file pone.0034207.s001.tif]

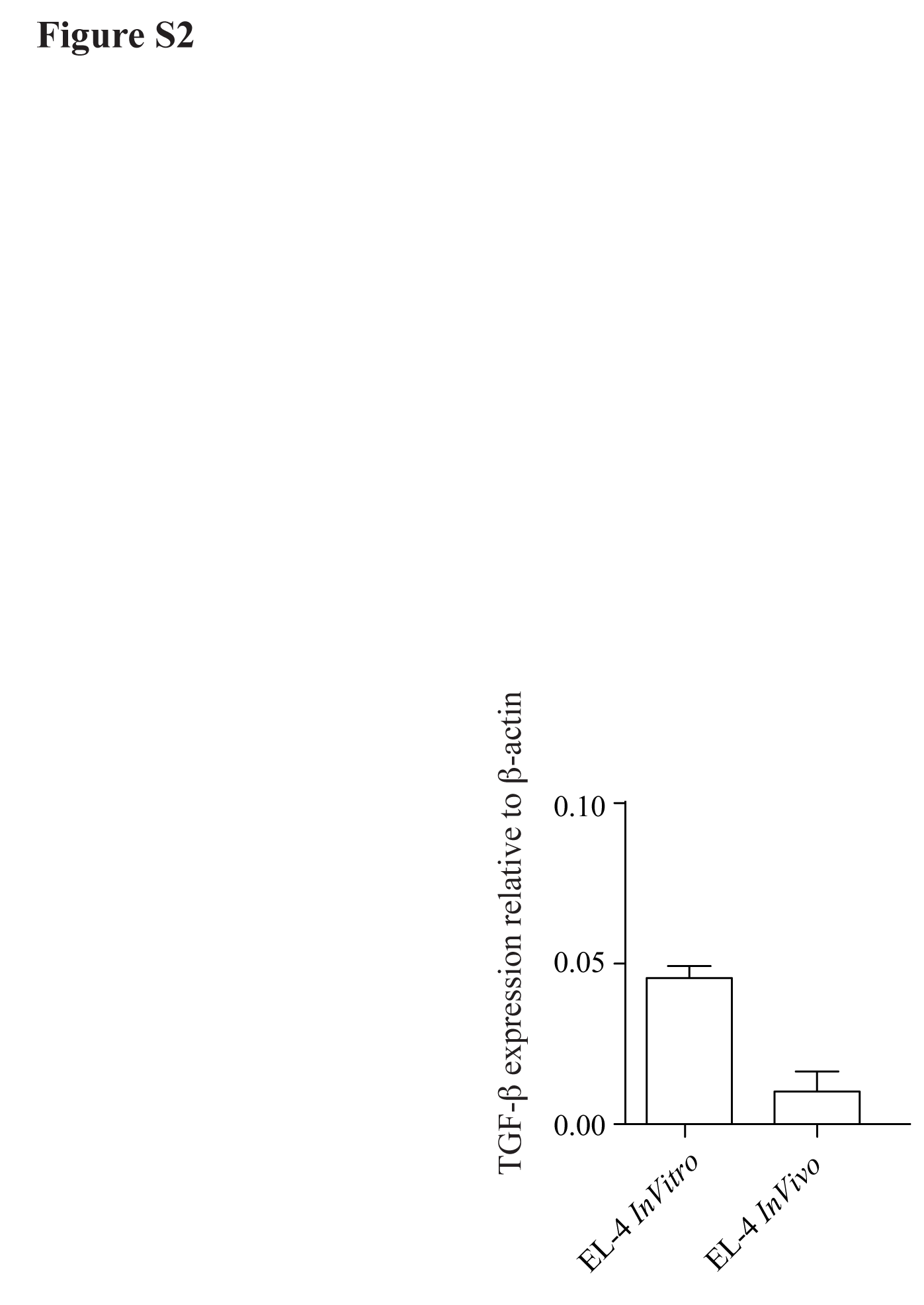

Supplement: Figure S2 — RNA from EL4 cells were analyzed with regard to TGFβ expression. CD3 expressing (MACS purification) cells from collagenase treated tumors were used as a source for EL4 in vivo RNA. (TIF) [file pone.0034207.s002.tif]

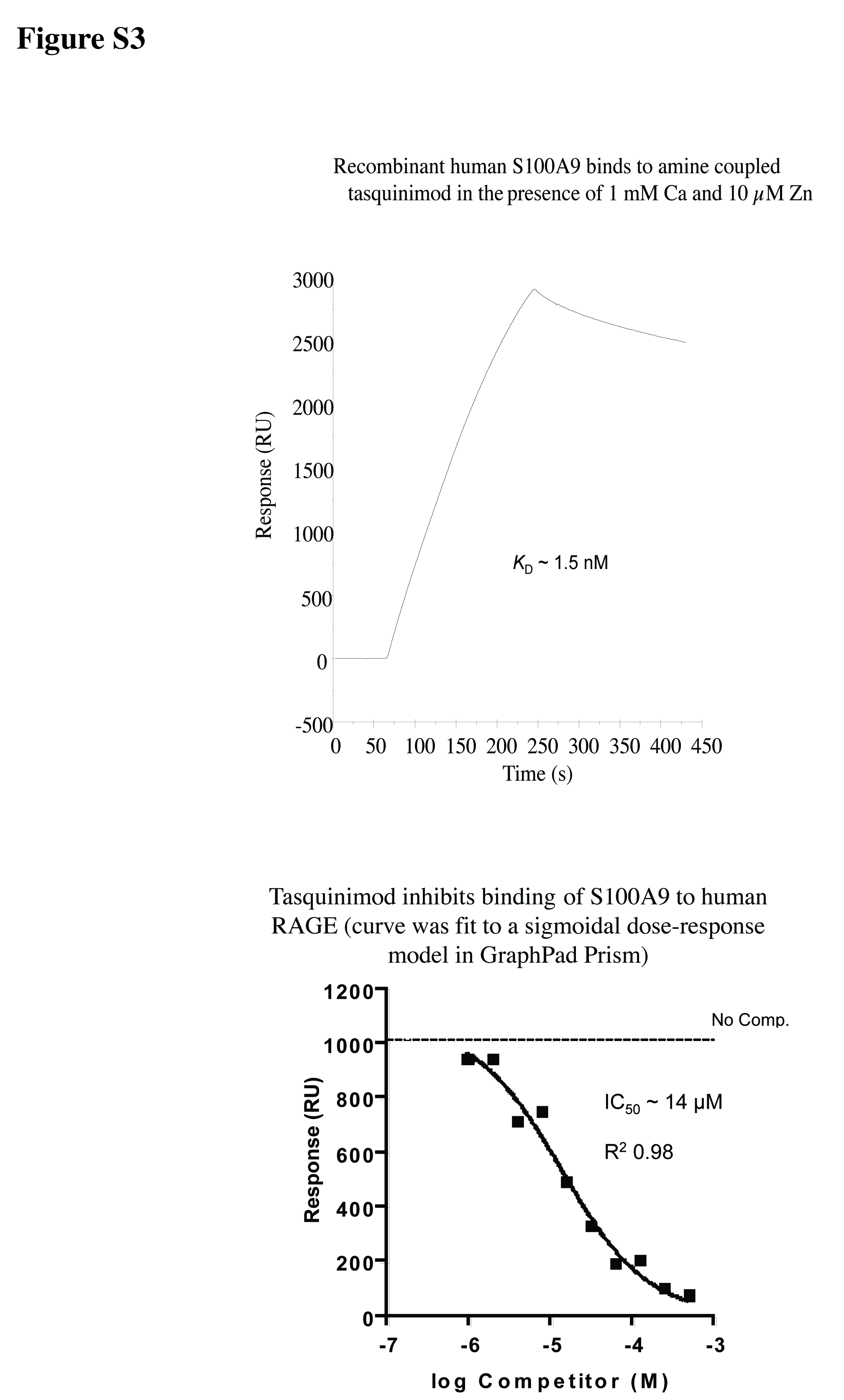

Supplement: Figure S3 — Upper panel: Recombinant S100A9 bind to amine coupled ABR-215050 in the presence of 1 mM Ca++ and 10 mM Zn++. Lower panel: ABR215050 inhibits the interaction between S100A9 and RAGE. (curve was fit to a sigmoidal dose-response model in GraphPad Prism). (TIF) [file pone.0034207.s003.tif]

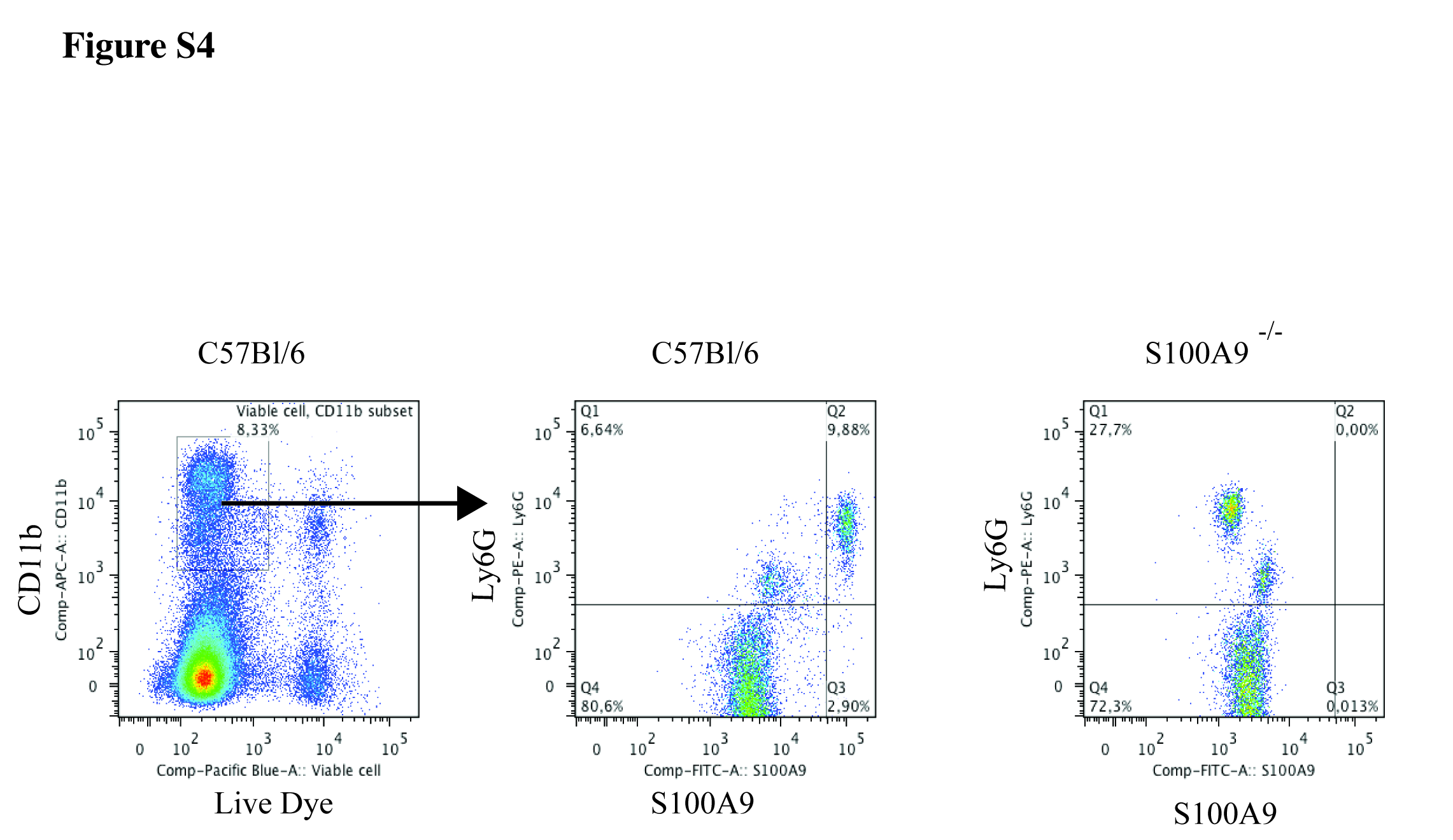

Supplement: Figure S4 — Intra-cellular staining of S100A9 in spleens from C57BL/6 and S100−/− mice. An affinity, purified rabbit anti-mouse S100A9 antibody was used to detect S100A9 in permeabilized cells. (TIF) [file pone.0034207.s004.tif]
